# Supplementary material for: Distinct D-box motifs in SPD-2 mediate APC/CFZR-1-dependent degradation and centrosomal localization in Caenorhabditis elegans embryos
Source: G3 (Bethesda). 2026 Mar 25;16(6):jkag071. doi: 10.1093/g3journal/jkag071 (PMC13232524; doi:10.1093/g3journal/jkag071)
Supplement: jkag071_Supplementary_Data [file jkag071_supplementary_data.pdf]

Supplementary Table 1. List of *C. elegans* Strains

Strain	Genotype	Origin
**N2**	*wild-type*	CGC
**OC14**	*zyg-1(it25[ZYG-1^{P442L}]) II*	O'Connell et al., 2001
**OC201**	*zyg-1(it25) fzr-1(bs31) II*	Kemp et al., 2007
**MTU6**	*fzr-1(bs31) II*	Medley et al., 2017
**MTU7**	*mat-3(or180) III*	Golden et al., 2000
**MTU490**	*spd-2(mhs650[SPD-2$^{DB1(2A)}$]) I; dpy-10(cn64)/+ II*	This study
**MTU686**	*spd-2(mhs720[SPD-2$^{DB2(2A)}$]) I; dpy-10(cn64)/+ II*	This study
**MTU493**	*spd-2(mhs653[SPD-2$^{DB3(2A)}$]) I; dpy-10(cn64)/+ II*	This study
**MTU696**	*spd-2(mhs651[SPD-2$^{DB1(2A)}$]) I; zyg-1(it25) II*	This study
**MTU689**	*spd-2(mhs720[SPD-2$^{DB2(2A)}$]) I; zyg-1(it25) II*	This study
**MTU546**	*spd-2(mhs653[SPD-2$^{DB3(2A)}$]) I; zyg-1(it25) II*	This study
**MTU337**	*spd-2(mhs580[SPD-2::2xHA]) I; dpy-10(cn64)/+ II*	Medley et al., 2021
**MTU369**	*fzr-1(mhs470[Ollas::FZR-1]) II*	Medley et al., 2021
**MTU375**	*spd-2(mhs580[SPD-2::2xHA]) I; zyg-1(it25) II*	This study
**MTU705**	*spd-2(mhs654mhs731[SPD-2$^{DB2+DB3(4A)}$]) I*	This study
**MTU806**	*spd-2(mhs580[SPD-2::2xHA]) I; fzr-1(mhs470[Ollas::FZR-1]) II*	This study
**MTU816**	*spd-2(mhs580[SPD-2::2xHA]) I; fzr-1(bs31) II*	This study
**MTU822**	*spd-2(mhs650mhs770[SPD-2$^{DB1(2A)}$::2xHA]) I*	This study
**MTU826**	*spd-2(mhs720mhs774[SPD-2$^{DB2(2A)}$::2xHA]) I*	This study
**MTU829**	*spd-2(mhs653mhs777[SPD-2$^{DB3(2A)}$::2xHA]) I*	This study

Supplementary Table 2. List of crRNA for CRISPR/Cas9 Genome

Gene	Target	Sequence (5’-3’)
dpy-10 (Arribere et al., 2014)	*dpy-10(cn64)* co-CRISPR	UUCUGCUGUCUUGAUUGACG
spd-2	C-terminus	UCUAUUCGAAAAUCUUGUAU
spd-2	D-box1	AUGGUUCGAAGAGUGCUCAA
spd-2	D-box2	UCGAACAAGACACUGACGAU
spd-2	D-box3	CUCCAGGAAGAAUACGUCGC
spd-2	D-box4, 5	UCAAAUUAACAAUCGAAGUC

Supplementary Table 3. List of ssODN Homologous Repair Templates for CRISPR/Cas9 Genome Editing

Gene	Variation	Sequence (5'-3')
dpy-10 (Arribere et al., 2014)	*dpy-10(cn64)*	CACTTGAACTTCAATACGGCAAGATGAGAATGACT<br>GGAAACCGTACCGCATGCGGTGCCTATGGTAGCG<br>GAGCTTCACATGGCTTCAGACCAACAGCCTAT
spd-2	C-terminal 2xHA Tag	AATCAGACATTTGTCAACGACGTTACAATTGTTCC<br>GAATACAAGATTTTCGAATAGAAAGGGAGGTTCC<br>GGTGGATCTGGTGGATCCTACCCATACGATGTTC<br>CAGATTACGCTTATCCATATGATGTTCCAGATTAT<br>GCTTAAAATCTTAACCTAACTTTCCAAATATTCTCT<br>G
spd-2	D-box1(AxxA)	TGATGACAAATCAAACTATTAATGAGTCAATGGTT<br>GCTCGCGTCGCTAAAGGAAACAATAAGAATCAAG<br>ATCTGTTCGCAGCATTAG
spd-2	D-box2(AxxA)	TTGAGAGTTGAAGTCGAAGTGGAAAACATTTCCGA<br>TGCTCAGTGTGCTGTTCGAGCTAGTACGGATAGT<br>ACCACACCAGTTTATCAAATCCT
spd-2	D-box3(AxxA)	TCTTCGCTTTCGACAGCCTCAACAACATCCTCATT<br>CGAGGCTCGTATTGCTCCTGGAGCCAAATTCTTT<br>GTGCATGTCGTTTGGGG
spd-2	D-box4(AxxA)	TCTTTGTGCATGTCGTTTGGGGTGAAGAAACCATG<br>GCTACTAGAGCTCGATTGTTAATTTGAATACAGAT<br>GAAATGGTATT
spd-2	D-box5(AxxA)	GTGCATGTCGTTTGGGGTGAAGAAACCATGCGAA<br>CTGCTCTTCGAGCGTTAATTTGAATACAGATGAAA<br>TGGTATTACATTT

Supplementary Figures

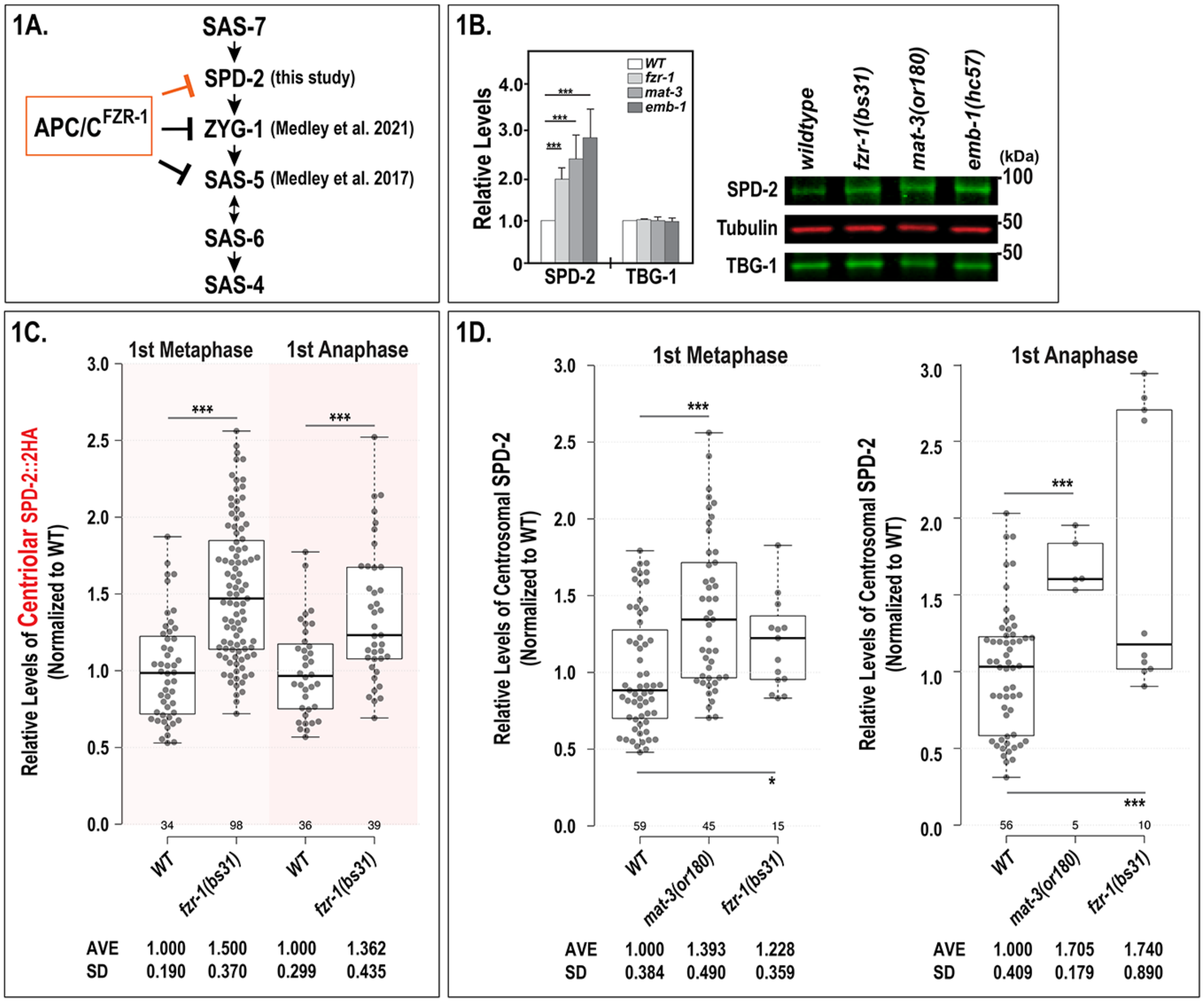

Supplementary Figure 1. (**A**) APC/C[FZR-1]-mediated proteolysis negatively regulates multiple centrosome factors *in C. elegans* embryos. (**B**) Overall SPD-2 levels are more than 2-fold higher in *fzr-1(bs31), mat-3(or180),* and *emb-1(hc57)* mutants. In contrast, TBG-1 levels remain unchanged across the strains. Representative Western blot with anti-SPD-2 and anti-TBG-1. Tubulin was used as a loading control. Average values are presented, and error bars are SD (*n*=8-10). ****p*<0.001 (two-tailed t-tests). (**C**) Quantification of centriolar SPD-2::2xHA levels in *fzr-1(bs31)* mutant embryos and WT controls at the first metaphase and anaphase. (**D**) Like *fzr-1(bs31)* mutants, *mat-3(or188)* mutant embryos exhibit elevated levels of total centrosomal SPD-2::2xHA during the first mitosis compared to wildtype (WT) controls. (**C-D**) Each dot represents a centrosome. In the plots, the box ranges from the first through the third quartile of the data. The thick bar indicates the median. The solid line extends 1.5 times the interquartile range or to the minimum and maximum data points. **p*<0.05*,* ***p*<0.01*,* ****p*<0.001 (two-tailed t-tests).

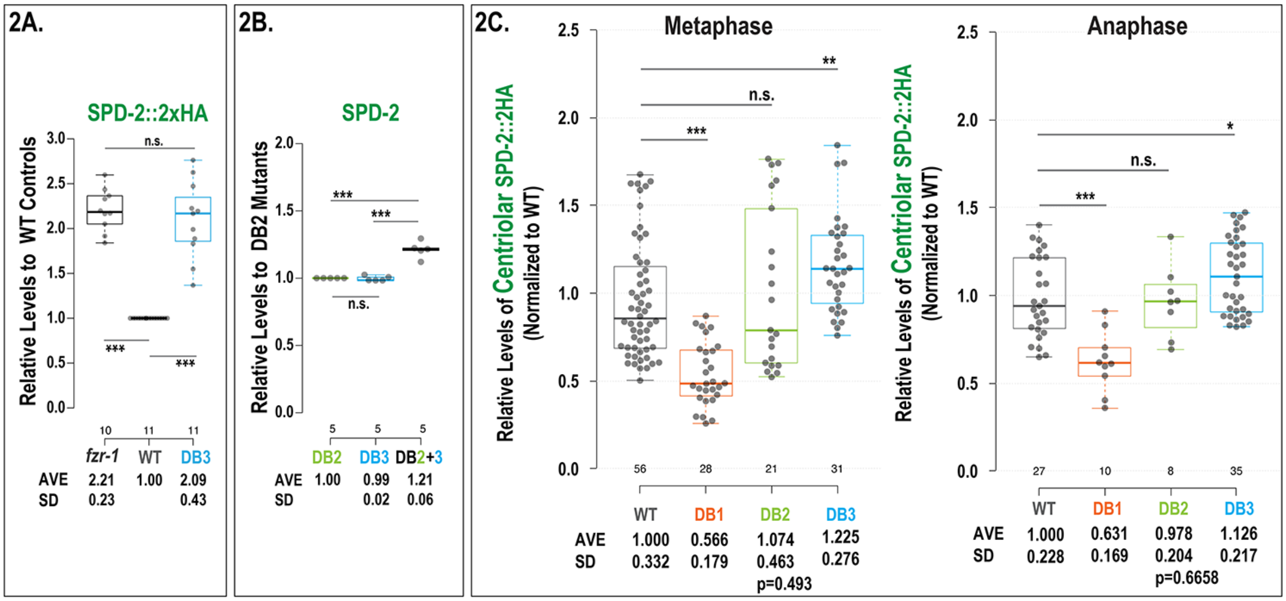

Supplementary Figure 2: (A) Quantitative Western blot analyses with anti-HA on *fzr-1(bs31)* mutant, WT control, and SPD-2:DB3(2A) mutant embryos expressing SPD-2::2xHA. **(B)** Quantitative Western blot analyses with anti-SPD-2 on SPD-2:DB2(2A), SPD-2:DB3(2A), and SPD-2:DB2+3(4A)mutant embryos. (**C**) Quantification of centriolar SPD-2::2xHA levels in SPD-2 degron mutants and WT controls at the first metaphase and anaphase. Each dot represents a centrosome. Average values are presented, and error bars are SD. In the plots, the box ranges from the first through the third quartile of the data. The thick bar indicates the median. The solid line extends 1.5 times the interquartile range, or to the minimum and maximum data points. $^{ns}p>0.05$, $^{*}p<0.05$, $^{**}p<0.01$, $^{***}p<0.001$ (two-tailed t-tests).

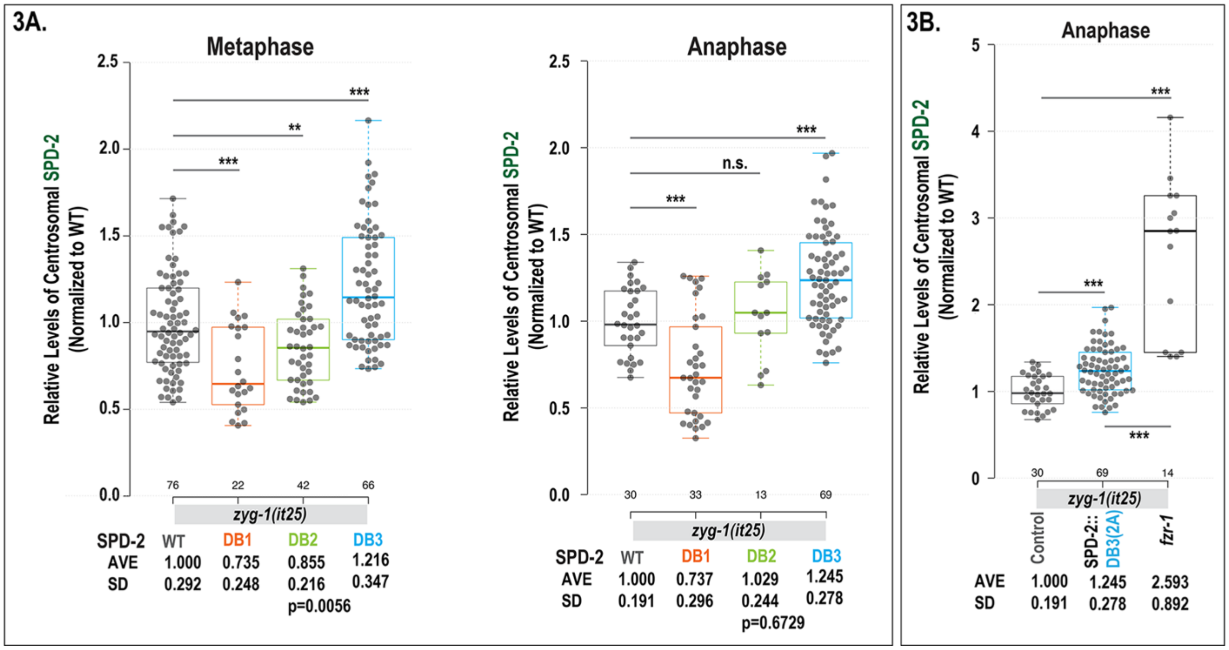

Supplementary Figure 3. (**A**) Quantification of total centromal SPD-2::2xHA levels during the first mitosis in *zyg-1(it25)* embryos carrying individual SPD-2 degron mutations or WT controls. (**B**) Quantification of total centrosomal SPD-2::2xHA levels in *zyg-1(it25)II* controls, *SPD-2::DB3(2A)I; zyg-1(it25)II* mutants, and *zyg-1(it25) fzr-1(bs31)II* embryos at the first anaphase. Each dot represents a centrosome. In the plots, the box ranges from the first through the third quartile of the data. The thick bar indicates the median. The solid line extends 1.5 times the interquartile range or to the minimum and maximum data point. $^{ns}p>0.05$, $^{**}p<0.01$, $^{***}p<0.001$ (two-tailed t-tests).
